# Midterm Prognosis of Sexagenary Patients after Transcatheter Device Closure of Atrial Septal Defects: a Single-Chinese Center Experience

**DOI:** 10.21470/1678-9741-2020-0387

**Published:** 2022

**Authors:** Kai-Peng Sun, Ning Xu, Shu-Ting Huang, Hua Cao, Qiang Chen

**Affiliations:** 1 Department of Cardiac Surgery, Fujian Maternity and Child Health Hospital, Affiliated Hospital of Fujian Medical University, Fuzhou, People’s Republic of China.; 2 Fujian Key Laboratory of Women and Children’s Critical Diseases Research, Fujian Maternity and Child Health Hospital, Fuzhou, People’s Republic of China.; 3 Department of Cardiovascular Surgery, Union Hospital, Fujian Medical University, Fuzhou, People’s Republic of China.

**Keywords:** Quality of Life, Sexagenary, Heart Septal Defects, Atrial, Prognosis, Feedback, Follow-Up Studies, Health Surveys

## Abstract

**Introduction:**

The objective of this study is to evaluate the efficacy and midterm prognosis of transcatheter device closure of atrial septal defects (ASDs) in sexagenary patients in China.

**Methods:**

Forty-six sexagenary patients who underwent transcatheter device closure of ASDs in our hospital were included in this study. The patients’ preoperative and postoperative clinical symptoms, echocardiographic results, and quality of life were investigated and analyzed.

**Results:**

Of the 46 sexagenary patients who participated in the study, 40 completed the study. After ASD closure, the clinical symptoms of the patients significantly improved, and the number of patients with dyspnea and palpitations significantly decreased after the operation. According to the echocardiographic results, few patients had a tiny residual shunt after closure, but the shunt disappeared completely at the three-month follow-up. The size of the right ventricular cavity was significantly smaller postoperatively compared with preoperatively. Regarding the patients’ quality of life, their feedback in all dimensions of the 36-Item Short-Form Health Survey (or SF-36) was significantly improved at the three-month follow-up, and it remained improved at the one-year follow-up.

**Conclusion:**

The clinical outcomes and subjective quality of life of sexagenary patients with ASDs improved significantly after transcatheter device closure of ASDs. Therefore, we believe that for sexagenary patients with ASDs, transcatheter device closure is a favorable treatment.

**Table:** 

Abbreviations, acronyms & symbols				
**ASD**	**= Atrial septal defect**		**RE**	**= Role emotional**
**BP**	**= Bodily pain**		**RP**	**= Role physical**
**E/A**	**= Peak of mitral flow spectrum**		**RV**	**= Right ventricle**
**GH**	**= General health**		**SD**	**= Standard deviation**
**LA**	**= Left atrium**		**SF**	**= Social functioning**
**LV**	**= Left ventricle**		**SF-36**	**= 36-Item Short-Form Health Survey**
**MH**	**= Mental health**		**TTE**	**= Transthoracic echocardiography**
**PF**	**= Physical functioning**		**VT**	**= Vitality**
**RA**	**= Right atrium**			

## INTRODUCTION

Patients with atrial septal defects (ASDs) often do not have any symptoms during their childhood or adolescence, and even those with large shunts between the interatrial septum may not have obvious symptoms in the early stage. With the progression of this disease, some symptoms caused by an excessive increase in the volume load of the right heart cavity and lung can develop. Some patients with large shunts have decreased exercise endurance, shortness of breath, and other symptoms, and eventually develop right heart failure. The correction of ASD can effectively inhibit the development of pulmonary hypertension, heart failure, arrhythmia, etc^[1]^. Among various treatment methods, transcatheter device closure of ASDs is widely used due to its advantages of faster physical recovery and less psychological and physical trauma, and its therapeutic effect has been confirmed in some studies^[2,3]^. Due to the age, physical condition, and other factors of sexagenary patients, there are still many factors worth exploring in the treatment of sexagenary patients with ASDs, particularly the prognosis after treatment. The purpose of this study was to evaluate the midterm prognosis of Chinese sexagenary patients who underwent transcatheter device closure of ASDs.

## METHODS

All research procedures were approved by the ethics committee of Fujian Medical University and were performed in accordance with the Helsinki declaration. The patients were informed of the study procedures and signed informed consent forms. The sample size was determined with the PASS 2020 Power Analysis and Sample Size Software (2020), NCSS, LLC, Kaysville, Utah, USA. The alpha value was set to be 0.05, and the power was set to be 0.90. Based on the calculation, the resulting minimum sample size was 39 patients. Considering a 15% drop rate, 46 patients were included in this study.

### Patients

Forty-six sexagenary patients who underwent transcatheter closure of ASDs in our cardiac center from January 2017 to January 2019 were selected. The exclusion criteria used to screen the patients were as follows: 1) patients with other congenital heart malformations who needed surgical correction, 2) patients with other acquired heart diseases, 3) patients with severe pulmonary hypertension or Eisenmenger’s syndrome, and 4) patients with severe diseases involving other organs. All patients were diagnosed with secundum ASDs by preoperative transthoracic echocardiography (TTE) with a significant left-to-right shunt. The patients’ demographic and clinical data are shown in Table 1. In the screening process, we excluded four patients who had developed Eisenmenger’s syndrome, which accounted for about 6% of sexagenary patients with ASDs during the same period, and 17 patients who underwent open heart surgical repair.

**Table 1:** Sociodemographic characteristics of the participants.

Item	Mean±SD/N (%)
Mean age (years)	64.8±3.7
Gender	
Male	14 (35%)
Female	26 (65%)
Weight (kg)	64.5±6.8
Height (cm)	162.3±5.6
ASD size (mm)	17.5±6.4
ASD occluder (mm)	22.4±10.6
In-hospital stay (days)	4.2±1.4
Diabetes mellitus	9 (22.5%)
Coronary heart disease	4 (10%)
Hypertension	12 (30%)
Dyslipidemia	15 (37.5%)
Smoking	10 (25%)

ASD=atrial septal defect; SD=standard deviation

### Treatment

The treatment was performed under local anesthesia, and a complete hemodynamic evaluation was done before the operation. During the operation, all procedures were performed under the monitoring of TTE. All patients underwent catheter examinations to determine the pulmonary artery pressure. The diameter of the ASD occluder was selected to be 4-6 mm larger than the ASD defect, as measured by TTE. No fenestrated occluder was used in this study. When the occluder was released into the defect, the stability and accuracy of its position were confirmed by TTE. The criteria for successful placement were that the occluder was placed firmly, and a tiny residual shunt was considered acceptable.

### Clinical Investigation

Based on the patients’ subjective knowledge of their own physical conditions, we recorded and assessed the symptoms of dyspnea and palpitations. The patients’ postoperative complications were investigated. Clinical tests were conducted upon admission and at the three-month and one-year follow-ups.

### Transthoracic Echocardiography

TTE was performed in the patients upon their admission and at the three-month and one-year follow-ups. The following echocardiographic parameters were recorded: 1) the inner diameter of the middle segment of the pulmonary artery, as measured with the short-axis view of the great vessels, 2) the length and width of the end-diastolic period of the right ventricle and the length and width of the end-systolic period of the right atrium, as measured by apical four-chamber endoscopy, 3) the left ventricular end-systolic and end-diastolic anteroposterior diameters, as measured using the left ventricular short-axial view, 4) the anteroposterior diameter of the middle end-diastolic period of the right ventricle, which was measured using the left ventricular parasternal long axis view, and 5) the left ventricular ejection fraction. It was important to measure the characteristics of the right ventricular cavity to evaluate the function of the right heart and determine the prognosis of treatment.

### Health-Related Quality of Life

The examination was conducted based on the subjective feelings of the patients about their own living conditions, and the Medical Outcomes Study 36-Item Short-From Health Survey (SF-36) was used. The questionnaire was mainly used to assess the physiological and social psychological conditions of patients regarding eight dimensions: 1. PF, physical functioning; 2. RP, role physical; 3. BP, bodily pain; 4. GH, general health; 5. VT, vitality; 6. SF, social functioning; 7. RE, role emotional; and 8. MH, mental health. The SF-36 is a general questionnaire that is widely used to assess quality of life. Each question had an exact score, and patients’ quality of life is positively correlated with the score. The survey was conducted upon the patients’ admission and at the three-month and one-year follow-ups. The investigation team was composed of two interventional cardiologists, one professional nurse, and one volunteer who was proficient in the local language. Since the survey results were mainly determined by the patients’ subjective feelings, the investigation team was allowed to help patients understand the questions but was not allowed to interfere with patients’ autonomy in answering them.

### Statistics

The IBM Corp. Released 2013, IBM SPSS Statistics for Windows, Version 22.0, Armonk, NY: IBM Corp. was used for statistical analysis. To analyze the preoperative and postoperative quality of life scores, the paired *t*-test was used, and the scores of various fields were positively correlated with patients' degree of satisfaction with their quality of life. The independent samples *t*-test was used to compare the preoperative and postoperative echocardiographic data. Continuous data are expressed as the mean ± standard deviation, and *P*<0.05 indicates a significant difference.

## RESULTS

The patients’ demographic and clinical data are shown in Table 1. Of the 46 sexagenary patients who participated in the study, 40 completed the study. Of the six patients who did not complete the study, three were relocated, and three were unable to complete the study due to a lack of time. The mean age of the patients who completed the study was 64.8±3.7 years, and the study population included 14 males and 26 females. The mean ASD size was 17.5±6.4 mm, and the mean length of in-hospital stay was 4.2±1.4 days.

**Fig. 1:**
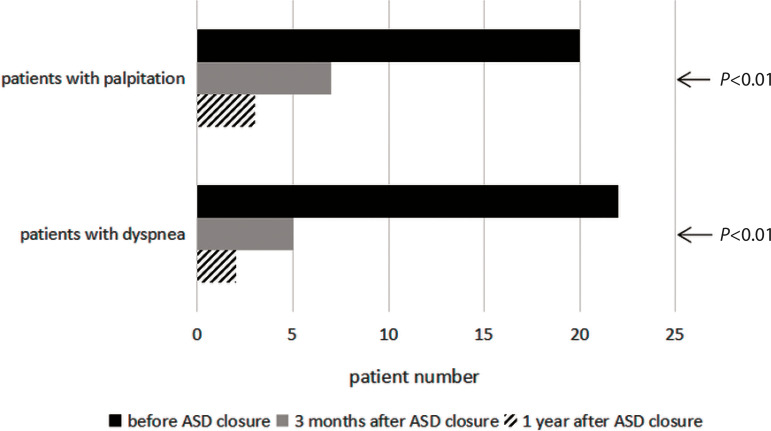
Number of patients with dyspnea or palpitations before and after atrial septal defect (ASD) device closure.

Figure 1 shows the number of patients with dyspnea or palpitations before and after ASD device closure. The postoperative clinical symptoms of the patients improved. The number of patients with dyspnea decreased from 22 (55.0%) preoperatively to five (12.5%) at three months after ASD closure and to only two (5.0%) at one year after ASD closure. The number of patients with palpitations decreased from 20 (50.0%) preoperatively to seven (17.5%) at three months after ASD closure and to only three (7.5%) at one year after ASD closure. The differences between the preoperative and postoperative values were statistically significant. None of the sexagenary patients who had completed the study had serious postoperative complications, such as occluder dislodgement, serious bleeding requiring reoperation, or cardiac tamponade. There was no sign of left heart failure immediately after ASD occlusion in all patients. Only three (7.5%) of the patients suffered from atrial fibrillation, and long-term anticoagulant therapy was recommended for these patients. However, in the follow-up investigation, all patients remained in good health, and no patients needed to be readmitted.

According to the preoperative catheter examination results, the average pulmonary artery pressure of the patients was 40±5.4 mmHg. Because significant left-to-right shunts are identified by TTE and mild-moderate pulmonary artery pressures are determined by direct catheter measurements, the average pulmonary vascular resistance and cardiac output are not routinely measured. On the echocardiographic findings, four (10.0%) of the patients were found to have a tiny residual shunt during the perioperative examination, but the shunt was completely resolved at the three-month reexamination. According to the analysis of the preoperative right ventricular cavity data, most of the patients had an enlarged right ventricular cavity, which tended to shrink after device closure of ASDs, and the detailed data are shown in Table 2.

**Table 2:** Changes of echocardiographic parameters after ASD closure.

Item	Preoperatively	3 months after ASD closure	1 year after ASD closure
End-systolic length of RA (mm)	56.8±8.4	45.7±5.9*	44.9±6.7*
End-systolic width of RA (mm)	49.5±7.9	37.6±8.3	36.8±7.3
End-diastolic anteroposterior diameter of RV (mm)	35.6±6.2	26.4±5.0*	24.2±6.2*
End-diastolic length of RV (mm)	72.0±10.5	62.2±11.2	61.4±11.7
End-diastolic width of RV (mm)	46.2±11.6	33.9±7.7*	32.4±8.8*
Inner diameter of the middle portion of the pulmonary artery (mm)	27.0±5.1	24.2±4.2*	22.6±6.1*
End-systolic anteroposterior diameter of LA (mm)	28.7±4.8	29.0±5.6	28.5±4.5
End-systolic anteroposterior diameter of LV (mm)	25.7±7.3	27.5±6.4	28.8±6.7
End-diastolic anteroposterior diameter of LV (mm)	41.2±5.0	45.8±6.2	46.3±5.8
E/A	1.4±0.3	1.3±0.4	1.3±0.2
Left ventricular ejection fraction (%)	61.3±5.8	60.5±6.7	59.5±6.2

ASD=atrial septal defect; E/A=peak of mitral flow spectrum; LA=left atrium; LV=left ventricle; RA=right atrium; RV=right ventricle

* Significantly different from preoperative value (*P*<0.05)

In addition to the improvements in patients’ clinical symptoms, the changes in the patients’ quality of life also caught our attention. Table 3 lists the SF-36 scores for the patients before and after ASD closure. The feedback of the patients in all dimensions improved significantly during the three-month follow-up period, and the feedback regarding quality of life still improved during the one-year follow-up period.

**Table 3:** Result of SF-36 scores before and after ASD closure.

	Before ASD closure	3 months after ASD closure	1 year after ASD closure	*P*-value before *vs.* 3 months after	*P*-value before *vs.* 1 year after
PF	50.2±15.4	62.6±10.1	64.2±9	*P*<0.001	*P*<0.001
RP	43.5±23.1	57.3±19.1	59.6±15.7	*P*<0.001	*P*=0.012
BP	58.6±18.4	68.3±11.4	68.7±10	*P*<0.001	*P*<0.001
GH	53.1±17.9	64.3±11.4	63.8±8.8	*P*<0.001	*P*<0.001
VT	49.0±19.5	61.0±10.9	61.8±7.7	*P*<0.001	*P*<0.001
SF	53.0±17.2	66.5±10.8	66.9±8.5	*P*<0.001	*P*<0.001
RE	41.5±24.3	56.4±25	58.5±22.9	*P*<0.001	*P*=0.012
MH	59.3±15.7	71.4±8.3	72.1±6.1	*P*<0.001	*P*<0.001

ASD=atrial septal defect; BP=bodily pain; GH=general health; MH=mental health; PF=physical functioning; RE=role emotional; RP=role physical; SF=social functioning; SF-36=36-Item Short-Form Health Survey; VT=vitality

## DISCUSSION

ASDs are common congenital heart diseases that are mainly treated by surgical repair in the early stage. Although the treatment results are satisfactory, this treatment is associated with some complications, such as arrhythmia, mental trauma, and psychological burden^[4,5]^. Since transcatheter closure of ASDs was developed in 1976, this technique has been improved and widely used as a routine treatment for more than 40 years^[6,7]^. There are still some cases of sexagenary patients with ASDs that are diagnosed in the clinic due to an imbalance in regional economic and cultural development and a lack of medical resources in China. Many sexagenary patients do not track or address their own symptoms, which is also a reason for this phenomenon^[8,9]^. Although open heart surgery can be performed safely and quickly in sexagenary patients, device closure seems to appeal more to them^[10,11]^. Compared with surgical repair, transcatheter device closure for sexagenary patients can prevent extracorporeal circulation-related complications and surgical trauma, as well as complications related to general anesthesia^[12,13]^. In the present study, many aspects of the postoperative recovery of sexagenary patients after treatment for ASDs were not studied.

Whether sexagenary patients with ASDs should undergo ASD closure is still controversial. Unlike younger patients, sexagenary patients are less likely to survive surgery and are more likely to have a shorter life span. Given the balance between therapeutic effectiveness and surgical risk, many sexagenary patients tend to vacillate between radical and conservative treatment. Therefore, the degree of improvement in cardiac function and quality of life of sexagenary patients after treatment has become an important factor influencing the choice of treatment plan. As transcatheter device closure of ASDs has been shown to be a safe and effective treatment in many studies, sexagenary patients have begun to prefer this treatment to prevent secondary structural changes in the heart due to an increased right ventricular volume load and to prevent the development of terminal heart failure^[14,15]^.

In this study, some of the sexagenary patients involved had clinical symptoms and changes in the structure of the right heart, while after ASD closure, the clinical symptoms of these patients improved. The number of patients with palpitations or dyspnea significantly decreased postoperatively compared with preoperatively. During the period of hospitalization, there were no serious complications. Only a small number of patients had some short-term and minor complications, which was consistent with the results reported in some studies^[16]^. During the short-term follow-up period, no patients needed to be readmitted due to cardiac symptoms. During the one-year follow-up period, all patients survived and had good health. Praz et al. concluded that it is safe and feasible to perform device closure of ASD in patients aged more than 60 years^[17]^. Their result also showed the same trend as ours. In our study, atrial fibrillation occurred in three patients. Research by Kuwata et al.^[18]^ suggested that left atrial appendage closure in ASD occlusion might be safe and feasible for the prevention of atrial fibrillation. We agreed with this view and it was worth recommending. However, due to the underdeveloped economic and intellectual level of our regions, it was not possible to perform two procedures at the same time in this study. For these patients, only device closure was completed, and long-term anticoagulant therapy was available.

In the treatment of sexagenary patients with ASD, the effect of hypertension, coronary heart disease, diabetes, and other diseases often has an important impact on clinical treatment. Studies have pointed out that in the treatment of these patients, the resulting complications are often an important factor affecting the treatment result, so the preoperative treatment of patients with the abovementioned diseases is also an important factor that needs to be addressed^[19]^. In the study by Jain et al., the presence of heart-related complications, such as atrial fibrillation and pulmonary hypertension, often complicated the treatment of ASDs^[20]^.

Studies in the literature have shown that after transcatheter device closure, the structure and function of the heart in patients significantly changed during the follow-up period, which was consistent with the results of our study^[21,22]^. With the disappearance of the left-to-right shunt, the volume load of the right cardiac cavity decreased, and the reduction of the cardiac burden indirectly led to an improvement in the patients’ clinical situation. According to the postoperative echocardiography results, the enlargement of the right ventricular cavity caused by the long-term presence of the left-to-right shunt improved to some extent, and the size of the right ventricular cavity also decreased.

As the cure rate of diseases continues to improve, patients are not only focused on resolving the diseases that affect them, but they are also focused on improving their quality of life during and after the treatment of the diseases. Conducting more research on patients’ quality of life can also be helpful in selecting patients’ treatment options. In this study, the SF-36 standard scale was adopted to assess quality of life, as it has been widely used in many studies and has been suggested to be very reliable^[23-26]^. According to our results, the scores for all dimensions of preoperative quality of life of the sexagenary patients were not high, but most patients’ feedback on their own physiological state showed that their quality of life significantly improved postoperatively at the short-term follow-up. In many existing studies, the focus of postoperative recovery has been mainly on the postoperative survival rate and physical function recovery, while patients’ psychological and social functions are still relatively unknown^[27,28]^.

However, the reason patients decide to pursue radical treatment is often not only for the pursuit of a longer life expectancy, but also to increase their level of comfort in daily life and reduce the limitations caused by the disease. For children, the effects of illnesses may be limited to physical discomfort, while for sexagenary patients, the adverse effects of illness on quality of life are not limited to the clinical symptoms. For long-term illnesses, the adverse effects of a disease on an individual’s ability to perform social activities, such as social interactions, employment, and physical activity, have also become important factors affecting the quality of life of patients. In this study, the results showed that the emotional and psychosocial domains of the patients’ quality of life improved significantly postoperatively compared with the preoperative period. In a study by Komar et al.^[29]^, it was also pointed out that the quality of life of sexagenary patients improved significantly after treatment compared to before treatment. It was found that most of the patients involved in the study were very concerned about whether there were ways to improve their physical function and quality of life in terms of their diet and level of physical activity after ASD closure. Therefore, it may be worth conducting research studies in the future to identify postoperative physical rehabilitation activities that can improve physical function and quality of life for sexagenary patients with ASDs.

### Limitations

Our study still has some limitations: 1) this is a retrospective study; 2) this is a single-center study, the experience of the surgeons might have geographical limitations, and this study only included short-term follow-ups, so the results of longer-term follow-ups remain to be explored; 3) the sample size was relatively small, so the ability to explore factors influencing the experimental results was decreased.

## CONCLUSION

In our study, the objective clinical conditions and the subjective quality of life of sexagenary patients who underwent transcatheter device closure of ASDs showed good improvement. Therefore, we believe that for sexagenary patients with ASDs, transcatheter device ASD closure is a beneficial treatment that can not only improve physical function but also help patients to return to their daily life.

**Table:** 

Authors' roles & responsibilities	
K-PS	Substantial contributions to the design of the work; analysis of data for the work; drafting the work; final approval of the version to be published
NX	Substantial contributions to the design of the work; analysis of data for the work; drafting the work
S-TH	Analysis of data for the work; final approval of the version to be published
HC	Acquisition of data for the work; final approval of the version to be published
QC	Substantial contributions to the design of the work; acquisition and analysis of data for the work; drafting the work; final approval of the version to be published
